# Characteristics of chest pain among children presenting to the pediatric emergency department

**DOI:** 10.25122/jml-2023-0280

**Published:** 2023-11

**Authors:** Abdulrahman Ahmad Alnaim, Hasna Wafi AlGarni, Hussain Adil Al Ghadeer, Muteb Abdulrahman Almulhim, Khalid Ibrahim Al Noaim, Mohammed Ahmad Al Ghamdi, Abdulaziz Abdullah Alahmari, Zainab Hejji Al Alawi, Muneera Abdulrahman Alabdulqader, Manal Mustafa Alghazal, Ohud Yousef Alhamad, Ahmed Eissa AlEissa, Ali Tawfiq AlAmer

**Affiliations:** 1 Department of Pediatrics, College of Medicine, King Faisal University, AlAhsa, Saudi Arabia; 2 Department of Pediatrics, Maternity and Children Hospital, Eastern Health Cluster, Dammam, Saudi Arabia; 3 Department of Pediatrics, Maternity and Children Hospital, AlAhsa Health Cluster, Saudi Arabia; 4 Department of Pediatrics, King Fahd Hospital, College of Medicine, Imam Abdulrahman Bin Faisal University, Dammam, Saudi Arabia

**Keywords:** Chest pain, cardiac, emergency department, nontraumatic, children, Saudi Arabia

## Abstract

Chest pain in pediatric patients is a common concern in pediatric emergency departments (ED). In most cases, benign conditions are related to noncardiac causes, and only a minority of the cases are caused by heart disease. This research aimed to evaluate the causes and characteristics of chest pain among children in a pediatric emergency department. This retrospective study evaluated children younger than 14 years of age who presented to the emergency department of a general pediatric hospital in the Eastern area of Saudi Arabia with non-traumatic chest pain between 2017 and 2022. The data included socioeconomic information, physical examination findings, and the results of basic investigations, such as chest X-ray and electrocardiogram. The Chi-square test was performed to compare various etiologies, with a 5% significant level. The study evaluated 310 patients with a mean age of 9.1±2.7 years. The majority of children presenting with chest pain had normal physical examinations, except 3.3% who showed respiratory and cardiac findings. The diagnostic tests indicated pneumonia in 2.9% and arrhythmia in 2.1% of children. Most patients were discharged with a diagnosis of idiopathic or muscular chest pain. The majority of patients (95%) were treated symptomatically in outpatient settings, with just one patient requiring hospitalization. The most common cause of chest pain prompting a child to visit the ED was idiopathic chest pain. Therefore, this study highlights the significance of obtaining a comprehensive medical history and physical examination to reveal important clues and help avoid unnecessary tests.

## INTRODUCTION

Chest pain is a common complaint in children and is commonly diagnosed and treated in the emergency department (ED). While cardiac-related causes are relatively uncommon in pediatric chest pain, they remain a primary concern for many parents, leading to a significant number of ED consultations [1-3]. Most cases are eventually attributed to idiopathic causes or self-limiting conditions such as costochondritis, psychogenic disorders, and pulmonary issues [4-7]. Despite this, the fear of serious cardiac problems is a common reason for parents to seek urgent medical attention for their children [8].

The way parents react to their child's pain can influence its perceived severity. For example, a parent's protective response to a child's chest pain may increase the anxiety of the child and thereby exacerbate the pain [9, 10]. Chest pain in children can also be caused by minor or severe trauma. In the emergency department, infants with chest pain only present with chest wall tenderness. However, it may be accompanied by additional cardiac symptoms such as palpitations, dyspnea, and vertigo. Children presenting to the ER with chest symptoms should undergo an electrocardiogram (ECG) and chest X-ray [11-13]. Patients may benefit from additional echocardiography, laboratory testing, and Holter monitoring [14, 15]. As numerous children present to the ED with idiopathic chest pain and endure numerous investigative tests, the current study aimed to determine the discharge diagnosis and inform about the testing methods. This will encourage medical professionals to conduct a thorough history and physical examination, which will disclose vital clues and prevent unnecessary tests.

## MATERIAL AND METHODS

Children younger than 14 years old who presented to the emergency department of a general pediatric hospital in the Eastern region of Saudi Arabia with non-traumatic chest symptoms were retrospectively evaluated between 2017 and 2022. The study evaluated demographic data (e.g., age, gender), comorbidities, clinical findings, chest radiography, and electrocardiogram (ECG). Children with traumatic chest pain, previously diagnosed with illness exacerbations, and younger than one year or older than 14 years were excluded from the study.

### Statistical analysis

The data were gathered, analyzed, and then input into Statistical Package for the Social Sciences, version 21 (SPSS: An IBM Company). All statistical tests were two-tailed with an alpha level of 0.05, and significance was determined if the p-value was less than or equal to 0.05. A descriptive analysis was conducted by prescribing frequency distributions and percentages for study variables such as the personal information and medical history of children. The Chi-square test, with a 5% significance level, was used to compare different etiologies of chest pain. Results of investigations were tabulated, and clinical findings and treatments were graphically represented.

## RESULTS

### Demographics and comorbidities

Table 1 presents the demographic characteristics and comorbidities of participants. A total of 310 children experiencing chest pain were included in the study. Children had a mean age of 9.1±2.7 years. There were 253 female children (81.6%) and 57 male children (18.4%). A significant portion, about 15.5%, had chronic diseases, with respiratory and hematological conditions being the most common. A total of 48 children (15.5%) had chronic diseases. Among these, the most common were respiratory conditions (33.2%), which included asthma and allergic rhinitis, hematological conditions (25.21%), which included sickle cell disease, glucose-6-phosphate dehydrogenase (G6PD) deficiency, iron deficiency, anemia, bleeding disorders, and others (42%), which included diabetes mellitus type I, gastroesophageal reflex, with no cardiovascular diseases reported.

**Table 1:** Demographic characteristics and comorbidities

Characteristics	N (%)
**Age (years)**	
Between 1 to 5	37 (11.9%)
Between 6-10	173 (55.8%)
Between 11-13	100 (32.3%)
**Gender**	
Male	57 (18.4%)
Female	253 (81.6%)
**Comorbidities**	
No	262 (84.5%)
Yes	48 (15.5%)
**Type of affection**	
Respiratory	16 (33.2%)
Hematological	12 (25.1%)
Cardiovascular	0 (0%)
Others	20 (42%)

The clinical evaluations revealed that most children (96.5%) had no abnormalities in their vital signs or clinical examinations (Table 2). Four patients had respiratory findings such as respiratory distress and diminished air entry. In seven cases, irregular heart rate, blood pressure, and murmur were detected.

**Table 2:** Vital signs and physical examination

Vital signs and physical examination	N (%)
**No abnormality detected**	**299 (96.5%)**
**Abnormality detected**	**11 (3.3%)**
**Respiratory**	**4 (1.2%)**
Distress	1 (0.3%)
Wheezing	1 (0.3%)
Decreased air entry	2 (0.6%)
**Cardiovascular**	**7 (2.1%)**
Tachycardia	2 (0.6%)
Bradycardia	1 (0.3%)
High blood pressure	1 (0.3%)
Irregular rhythm	1 (0.3%)
Murmur	2 (0.6%)

Basic evaluations, including electrocardiograms, chest X-rays, and the subsequent management of patients, are described in Table 3. All patients underwent an ECG and a chest X-ray as part of their evaluation. The findings from these evaluations revealed that pneumonia was the most common abnormality, identified in 2.6% of the cases, followed by a prolonged QT interval (N=2), pericarditis with effusion, right bundle branch block (RBBB), and Wolff-Parkinson-White (WPW). There were no particular findings in 95.5% of cases (N=296). Out of the sample, 297 patients (95.8%) received symptomatic therapy, seven (2.3%) received azithromycin, and other sporadic therapies were administered per case, including Propranolol and pericardiocentesis. Only one case was hospitalized at the critical care unit.

**Table 3:** Overview of diagnostic findings and management in pediatric chest pain cases

	N (%)
**No abnormality detected**	**296 (95.5%)**
**Abnormality detected**	**14 (4.4%)**
**Chest X-ray findings**	**9 (2.9%)**
Pneumonia	8 (2.6%)
Acute chest syndrome	1 (0.3%)
**Electrocardiogram**	**5 (1.5%)**
Prolonged QT interval	2 (0.6%)
Pericarditis	1 (0.3%)
Right bundle branch block	1 (0.3%)
Wolff-Parkinson-White (WPW)	1 (0.3%)
**Management**	
Symptomatic	297 (95.8%)
Pharmacological (antibiotic, beta blocker)	10 (2.8%)
Non-pharmacological	1 (0.3%)

Table 4 highlights the basic evaluations concerning the comorbidities and clinical findings. This table shows that 96.2% of children without chronic illness had fewer aberrant findings than those with chronic diseases. Among children with a normal physical examination, the basic workup revealed no abnormalities in 96.3% of cases, compared to cardiac and respiratory findings in 71.4% and 75%, respectively.

**Table 4:** Basic diagnostic evaluations in relation to comorbidities and clinical findings

Variable	ECG & X-ray		
	Unremarkable N (%)	Cardiac findings N (%)	Respiratory findings N (%)
**Chronic diseases**			
Yes	44 (91.7%)	2 (4.2%)	2 (4.2%)
No	252 (96.2%)	4 (1.5%)	6 (2.3%)
**Physical examination**			
No abnormality detected	288 (96.3%)	3 (1%)	8 (2.7%)
**Cardiac findings**	**5 (71.4%)**	**2 (28.6%)**	**0**
**Respiratory findings**	**3 (75%)**	**1 (25%)**	**0**

Table 5 reveals the factors associated with positive findings following basic evaluations. Only clinical examination findings were substantially correlated with investigation findings, with 28.6% of cardiac-related patients on examination showing positive investigations compared to 3.7% of unremarkable examination findings and 25% of those with respiratory-related symptoms (p-value=.001).

**Table 5:** Predictive factors of positive findings during chest pain investigations

Variable	ECG & X-ray		p-value
	Abnormal	Normal	
	N (%)	N (%)	
**Age (years)**			0.278
1-5	2 (5.4%)	35 (94.6%)	
6-10	5 (2.9%)	168 (97.1%)	
11-15	7 (7%)	93 (93%)	
**Gender**			0.764^EP^
Male	3 (5.3%)	54 (94.7%)	
Female	11 (4.3%)	242 (95.7%)	
**Physical examination**			0.001*^EP^
No abnormality detected	11 (3.7%)	288 (96.3%)	
Cardiac	2 (28.6%)	5 (71.4%)	
Respiratory	1 (25%)	3 (75%)	
**Comorbidities**			0.166
Yes	4 (8.3%)	44 (91.7%)	
No	10 (3.8%)	252 (96.2%)	

EP: Pearson X^2^ test; EP: Exact probability test; *p<0.05 (significant)

## DISCUSSION

As most young children and adolescents are unable to precisely describe their discomfort, diagnosing children at higher risk or ultimately ruling out a cardiac cause is challenging for clinicians [16]. This may be accompanied by unnecessary diagnostic investigations, testing, or consultations, resulting in a longer ED stay and increased costs. In most cases, a thorough patient history and physical examination should be sufficient for the correct management [17-20]. The purpose of the present study was to determine whether any children who presented to the emergency department with chest pain should undergo extensive investigation and imaging. The results revealed that only a small percentage of children with chest pain who visited the emergency department had a chronic health condition, with asthma being the most reported condition, followed by hematological disorders and obesity. Considering clinical examination, most children exhibited normal findings, with only seven cases demonstrating cardiovascular issues and four showing respiratory abnormalities. All children underwent an electrocardiogram and chest X-ray. Pneumonia was diagnosed in eight cases, and other specific conditions like prolonged QT interval, acute chest syndrome, pericarditis with effusion, RBBB, and WPW were rare, with one case diagnosed for each condition. The vast majority of investigations (95.5%; N=296) yielded unremarkable results.

Children with chest pain were most frequently prescribed oral analgesics in the emergency department. These results were consistent with most of the research on the topic. According to the literature, chest pain in pediatric ER is generally benign and rarely caused by significant cardiac or noncardiac diseases [21-23]. A study conducted by Pissarra R *et al*. [24] revealed that a cardiac etiology is reported among 1% of pediatric chest pain, with hospital admissions around 3%, while musculoskeletal was the most frequent cause, as reported in many studies [7, 19, 23].

Our research revealed that the majority of children with no chronic health problems showed negative investigation results, translating into a lack of need for further assessment. In contrast, a smaller percentage of those who had chronic health problems showed positive clinical investigation findings related to chronic diseases. The majority of children with normal clinical examination findings also had normal investigations. Conversely, it was observed that about three-fourths of the children who presented with suspected cardiac signs or respiratory symptoms had unremarkable results in their clinical investigations. This indicates that even among children with specific symptoms suggestive of more serious conditions, the likelihood of finding significant abnormalities through further testing is relatively low. This pattern aligns with the findings of Pissarra R *et al*. [24], who reported that a significant proportion of pediatric patients (62.8%) with chest pain had no notable findings during physical examinations.

Subsequent examinations revealed that a significant portion of children had musculoskeletal disorder-related chest pain or idiopathic pain. Another study found that out of 103 children who reported to the emergency room with chest pain, five had pneumonia, and three had pneumothorax. Furthermore, just four of them had ECG abnormalities, indicating no need for additional diagnostic investigations without substantial clinical symptoms and findings [2]. Another study [25] highlighted that medical history and physical examination are usually sufficient to rule out cardiac and other significant causes of chest pain among children. Additional assessments or referrals may be necessary in situations where the cause of the chest pain remains unclear or if specific related symptoms are identified. Furthermore, Sarder *et al*. [26] found that all pediatric patients with chest pain underwent ECGs and echocardiography, which revealed no underlying cardiac justification for the chest pain and demonstrated that none of the cases required cardiac interventions or therapy. In terms of investigation results concerning clinical examination and chronic diseases to determine whether there is a need for further investigation among children with positive clinical assessment findings, the study found that most children with no chronic health problem had good investigation results, indicating no need for further assessment, while a lower percentage of those with chronic health problems had positive clinical investigation findings. Moreover, most children who had an ordinary clinical examination also had unremarkable ECG and echocardiography findings. On the other hand, almost three-fourths of children with suspected cardiac signs had unremarkable clinical investigation results, similar to children with respiratory symptoms. Therefore, most children reporting chest discomfort with normal clinical examinations in the ER may not require additional supplemental diagnostic tests and investigations. Still, those with positive clinical examinations may if they have a history of chronic health problems. These findings were similar to those of Pissarra *et al*. [24], who discovered that 45.5% of those examined showed changes, with 62.8% having chest wall pain. The treatment approach for children presenting with chest discomfort in the emergency room primarily involved the administration of oral analgesics. These findings were consistent with the broader findings reported in the medical literature regarding the management of pediatric chest pain in emergency settings. According to the literature, chest discomfort in children who visit the ER is typically benign and rarely caused by significant cardiac or noncardiac disorders [21-23].

## CONCLUSION

Similar to previous research, our study concluded that it is unlikely to indicate a major underlying condition in the absence of concomitant symptoms such as dyspnea, cold sweats, exhaustion, intense chest pain, or long-term upper abdomen discomfort.

## Data Availability

Further data is available from the corresponding author upon reasonable request.
